# A fifth major genetic group among honeybees revealed in Syria

**DOI:** 10.1186/1471-2156-14-117

**Published:** 2013-12-06

**Authors:** Mohamed Alburaki, Bénédicte Bertrand, Hélène Legout, Sibyle Moulin, Ali Alburaki, Walter Steven Sheppard, Lionel Garnery

**Affiliations:** 1 CNRS, Laboratoire Evolution, Génomes et Spéciation LEGS, Avenue de la Terrasse, 91189 Gif-sur-Yvette, France; 2 Université Laval, Institut de Biologie Intégrative et des Systèmes (IBIS), Pavillon Charles-Eugène Marchand, 1030, Avenue de la Médecine, Québec, QC G1V 0A6, Canada; 3 Université de Versailles Saint-Quentin, Versailles, France; 4 University of Damascus, Faculty of Agriculture, PO BOX: 30621, Damascus, Syria; 5 Department of Entomology, Washington State University, Pollman, WA 99164-6382, USA

**Keywords:** *Apis mellifera syriaca*, Microsatellite, Conservation genetics, Population genetics

## Abstract

**Background:**

Apiculture has been practiced in North Africa and the Middle-East from antiquity. Several thousand years of selective breeding have left a mosaic of *Apis mellifera* subspecies in the Middle-East, many uniquely adapted and survived to local environmental conditions. In this study we explore the genetic diversity of *A. mellifera* from Syria (n = 1258), Lebanon (n = 169) and Iraq (n = 35) based on 14 short tandem repeat (STR) loci in the context of reference populations from throughout the Old World (n = 732).

**Results:**

Our data suggest that the Syrian honeybee *Apis mellifera syriaca* occurs in both Syrian and Lebanese territories, with no significant genetic variability between respective populations from Syria and Lebanon. All studied populations clustered within a new fifth independent nuclear cluster, congruent with an mtDNA Z haplotype identified in a previous study. Syrian honeybee populations are not associated with Oriental lineage O, except for sporadic introgression into some populations close to the Turkish and Iraqi borders. Southern Syrian and Lebanese populations demonstrated high levels of genetic diversity compared to the northern populations.

**Conclusion:**

This study revealed the effects of foreign queen importations on Syrian bee populations, especially for the region of Tartus, where extensive introgression of *A. m. anatolica* and/or *A. m. caucasica* alleles were identified. The policy of creating genetic conservation centers for the Syrian subspecies should take into consideration the influence of the oriental lineage O from the northern Syrian border and the large population of genetically divergent indigenous honeybees located in southern Syria.

## Background

Morphometric approaches subdivide the honeybee *Apis mellifera* L. into four different evolutionary lineages: West Mediterranean (M), which includes the Western European subspecies *Apis mellifera mellifera*; North Mediterranean (C); African lineage (A) which groups the African honeybee subspecies; and finally the Oriental lineage (O) located geographically in the Middle East and represented mainly by Caucasian and Turkish honeybees *A. m. caucasica* and *A. m. anatoliaca* respectively [1]. Several further subspecific divisions have been recognized within the aforementioned lineages via multivariate analysis of morphometrical characters [2]. Genetic studies have largely confirmed the morphometrical classification [3-7].

The Syrian honeybee*, A. m. syriaca*, occurs throughout most of Syria, Lebanon and the North Jordanian territories [1] and sits within a complex patchwork of interlocking populations. Morphometrically, *A. m. syriaca* belongs to the African lineage A [1,2] but has been grouped incongruently to the Oriental mitochondrial lineage O [8]. However, foreign queen introductions are known to result in mitochondrial introgression among evolutionary lineages [9,10]. As such, a more recent study based on mitochondrial DNA (mtDNA) variation, suggests that Syrian *A. m. syriaca* in fact belong to the African lineage A (mitochondrial haplotype subgroup Z), consistent with the morphometrical data [1,11]. Nonetheless *A. m. syriaca* from northern Syria are spatially indistinct from *A. m. anatoliaca* in Turkey, and a question remains concerning the level of introgression between them. Indeed, based on nuclear single-nucleotide polymorphism (SNP) markers [6], a number of Syrian honeybee samples sampled from the northern Syrian-Turkish border grouped with populations of *A. m. anatoliaca* and *A. m. caucasica*.

Ftayeh, 1994 [12] reported that Syrian honeybee populations sampled from two Syrian regions (Hassaka, Deir-Alzoor) close to Iraq retained morphometrical characteristics with closer resemblance *A. m. meda* (Iran) than to *A. m. syriaca*. The link between Iraqi, Iranian and Eastern Syrian individuals is consistent with mtDNA results reported by [11], whereby almost all samples coming from these areas grouped outside African mitochondrial lineage A.

The African mitochondrial lineage A, to which most African bees belong (as well as many *A. m. syriaca*), is known to be the most genetically diverse among the species [1-3,13-15]. Lineage A pervades throughout Africa. Nonetheless, some confusion exists around *A. m. lamarckii*, colonies from Egypt and Northern Sudan, on the southerly extent of range of *A. m. syriaca*. Franck, 2000 [8] classified *A. m. lamarckii* to a newly defined mitochondrial lineage called ‘O’. Not only did such a designation create confusion with Ruttner’s morphometric lineage (O), but [11] demonstrated, via the inclusion of further *A. m. lamarckii* and *A. m. syriaca* samples, that the former grouped among African populations [11,16].

Thus the Syrian honeybee population is located in a spatial contact zone between morphometrical lineages O from the north and north-east (*A. m. anatoliaca* and *A. m. meda*) and (A) (*A. m. syriaca*) from the south. The respective north–south gradient of the (C / O) and (A) mtDNA among honeybee populations from Syria in congruent with this pattern of diversification [11].

To develop coherent policies for the conservation of local honeybee races, it is first necessary to characterize the genetic diversity of honeybee races in their native areas. In this paper, the genetic variability of *Apis mellifera syriaca* from three different countries, Syria, Lebanon and Iraq is explored via a dataset of unprecedented size. We identify patterns of migration and introgression, including the effect of importation and management practices, finally showing that Syrian honeybees form a genetically distinct group that deserves immediate measures to promote its conservation.

## Results

### Gene diversity

**Figure 1:**
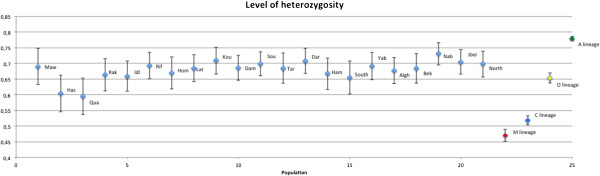
**Heterozygosity of overall loci of each studied populations as well as the four reference lineages M, A, C and O.** Refer to Table 1 for the abbreviations.

Mean expected heterozygosities per locus and over loci for all the studied populations (Table 1) are summarized in Figure 1, as well as in Additional file 1: Table S1. For Syrian, Lebanese and Iraqi populations heterozygosity ranged between (0.595-0.709), (0.684-0.731) and 0.690, respectively (Additional file 1: Table S1). In comparison, expected heterozygosity for overall loci concerning the reference populations ranged from 0.425 to 0.496 for M lineage populations, from 0.478 to 0.529 for the C lineage, from 0.612 to 0.672 for O lineage and from 0.757 to 0.811 for A lineage, Figure 1.

**Table 1:** Shows the number of honeybee workers sampled from each Syrian, Lebanon and Iraq region, the latitude, the longitude and the altitude of each sampled region

**Country / honeybee population**	**Abbreviation**	**Number of sample / honeybee worker**	**Latitude**	**Longitude**	**Altitude**
**Syria**					
Damascus	Dam	96	33.50 (33°30′00″N)	36.32 (36°19′12″E)	~730 m
Rif Dimashq	Rif	96	33,82 (33°48′34″N)	36,45 (36°29′18″E)	~1200 m
As-Suwayda	Sou	96	32.71 (32°42′36″N)	36.56 (36°33′36″E)	~900 m
Hama	Ham	100	35.15 (35°09′00″N)	36.73 (36°43′48″E)	~280 m
Daraa	Dar	96	32.63 (32°37′48″N)	36.10 (36°06′00″E)	~550 m
Al-Hasakah	Has	146	36.50 (36°30′00″N)	40.76 (40°45′36″E)	~310 m
Homs	Hom	73	34.73 (34°43′48″N)	36.72 (36°43′12″E)	~510 m
Idlib	Idl	96	35.94 (35°56′24″N)	36.63 (36°37′48″E)	~470 m
Quneitra	Kou	96	33.13 (33°07′48″N)	35.82 (35°49′12″E)	~990 m
Latakia	Lat	41	35.54 (35°32′24″N)	35.78 (35°46′48″E)	~30 m
Ar-Raqqah	Rak	96	35.93 (35°55′48″N)	39.02 (39°01′12″E)	~230 m
Tartus	Tar	40	34.90 (34°54′00″N)	35.89 (35°53′24″E)	~0 m
Al-Quamishli	Qua	50	36.50 (36°30′00″N)	40.76 (40°45′36″E)	~310 m
Yabroud	Yab	50	33,82 (33°48′34″N)	36,45 (36°29′18″E)	~1400 m
Alghab	Algh	50	35.15 (35°09′00″N)	36.73 (36°43′48″E)	~280 m
South	South	36	32.63 (32°37′48″N)	36.10 (36°06′00″E)	~550 m
**Lebanon**					
North	North	21	34.68(34°50′00″N)	35.92(35°80′00″E)	~800 m
Jbeil	Jbei	50	33.05(34°05′00″N)	35.86(35°39′00″E)	~250 m
Bekaa	Bek	48	35.18(34°10′00″N)	36.30(36°10′00″E)	~750 m
Nabatiyeh	Nab	50	33.52(33°23′00″N)	35.45(35°27′00″E)	~550 m
**Iraq**					
Al-Mawsil	Maw	35	36.30(36°45′12″N)	43.08(43°59′96″E)	**~**227 m
Total	-	1462	-	-	-

The average number of alleles detected in the Syrian, Lebanese and Iraqi populations ranged from 3 (A88) to 30 (A7). The highest averages of detected alleles overall loci were (12.36), (12.50) and (13.71) for populations of Rif-Dimashq, As-Suwayda and Quneitra, respectively (Additional file 1: Table S2).

### Population structure

**Figure 2:**
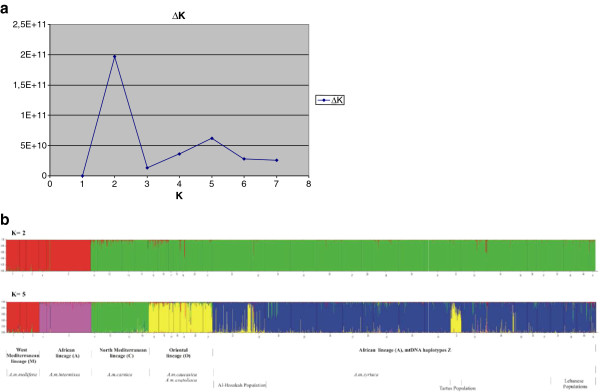
**Genetic clusters of Syrian, Lebanese and Iraqi honeybee populations using Structure program. a:** Identification of the number of genetic clusters in the 14 microsatellite dataset using the method of [17], K is the number of clusters. **b:** Genetic clustering of Syrian honeybee and other honeybee subspecies representing the four evolutionary lineages M, A, C and O, using 14 microsatellite loci and STRUCTURE program v 2.3.3. Bleu cluster is the analysed populations from Syria, Lebanon and Iraq, while the other colours are clusters of honeybee subspecies from the four evolutionary lineages.

STRUCTURE software was used to define genetic structure among our studied populations. The program was run for values of K = 1-9, while the most likely number of clusters K was calculated according to [17]. The highest value of ∆K was detected when a model assuming two populations was set (Figure 2a), however a secondary peak was observed at K = 5.

For *K* = 2, the first cluster includes populations from the West Mediterranean (M) and the African (A) lineages whilst the second cluster grouped all our studied populations with the populations belonging to the North Mediterranean (C) and Oriental (O) lineages. When five clusters are assumed (K = 5), referenced populations from each of the four evolutionary lineages (M, A, C and O) are grouped together and the populations from our study are then grouped in a fifth cluster. Within this fifth group, two populations (Al-Hasakah and Tartus) show clearly heterogeneity in their composition, combining individuals classified as belonging to the fifth cluster with a strong allelic introgression coming from the O lineage (Figure 2b).

In our first STRUCTURE analysis, Syrian samples were perhaps overrepresented. We undertook a second analysis to account for any bias introduced by population sample size imbalance. As such, 170 to 200 individuals were selected at random from the Syrian population and analysed alongside the reference populations. This process was repeated eight times. In each analysis, samples from the Syrian populations clustered consistently as a fifth group. Thus we can conclude that the fifth group - *Apis mellifera syriaca* – is not simply an artefact of increased sample size (Additional file 1: Figure S1).

Finally, DAPC, which we adopted as a secondary clustering approach, supported the delineation of nine population clusters. Interestingly, the distribution of these nine clusters in the resulting multidimensional scaling plot is consistent with the five clusters indicated via the secondary peak in ∆K for STRUCTURE, Additional file 1: Figure S2. We found no evidence of strong subdivision along the same lines as *K* = 2, but we did find good evidence for the presence of a distinct *Apis mellifera syriaca* group.

### Population-level divergence

**Figure 3:**
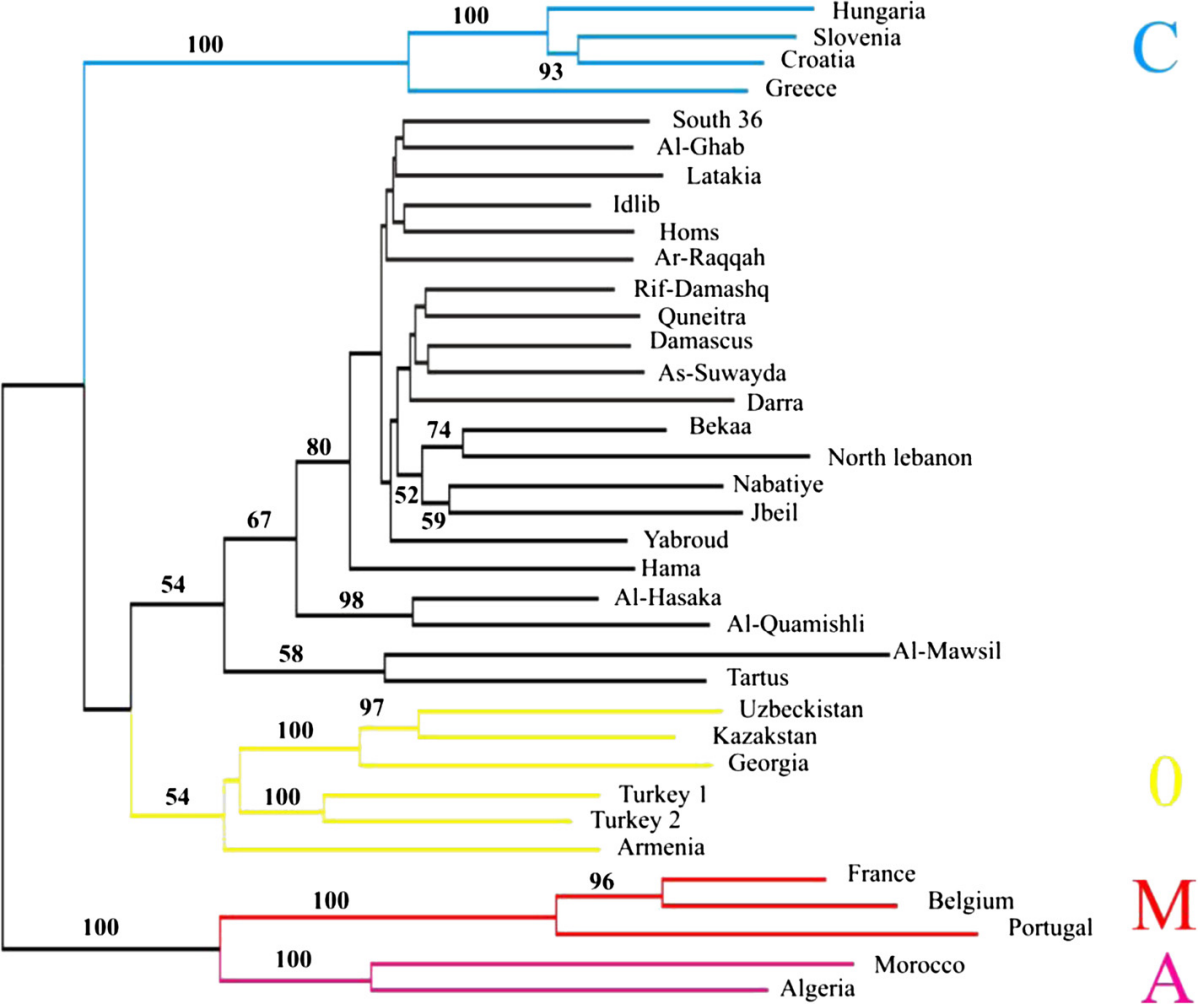
**A neighbour-joining tree calculated from microsatellite data of the population samples using the chord distance of [**[18]**].**

**Figure 4:**
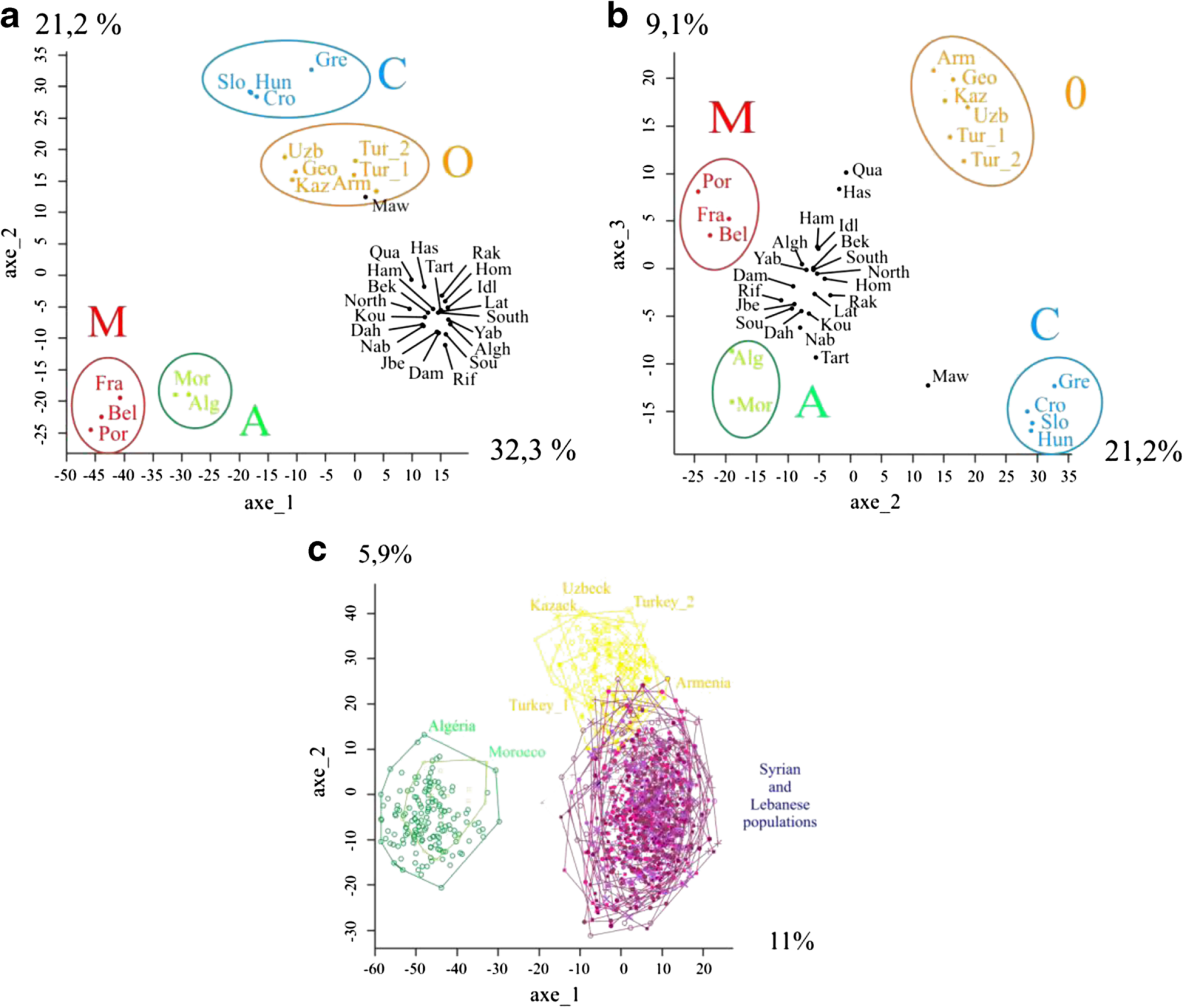
**Principal component analysis (PCoA) based on 14 microsatellite loci showing patterns of genetic variation in the studied populations. (a and b)** discriminate the four evolutionary lineages M, A, C and O on axes 1, 2 and 3. The studied populations cluster independently with both A and O lineages **(c)**.

Inter-population measures of genetic distance resulted in a neighbour-joining tree with a robustly supported topology (Figure 3). A, M and C lineages are strongly supported by long internal branches and robust bootstrap values (100%). Meanwhile, populations from Syria, Lebanon and Iraq cluster as a sister group to the O lineage (54% bootstrap). Populations from Tartus (Syria) and Al-Mawsil (Iraq) are outliers with respect to populations from our study area. A principal coordinates analysis (PCoA) is presented in Figure 4a, b and c supports the outlier status of Tartus and Al-Mawsil populations, as well as, to a lesser extent Al-Hasaka and Al-Quamishi. Like the NJ tree, the PCoA analysis broadly supports a division of major lineages C + O / M + A and, in combination, the three axes separate and clearly discriminate the four major evolutionary lineages M, A, C, and O. Crucially, the PCoA supports the delineation of isolates from our study area as a separate group, albeit related to O, with which it partially overlaps.

Pairwise inter-population *F*_ST_ values consistently support strong subdivision between Syrian/Lebanese populations and reference populations (M, *F*_ST_ = 0.31-0.42), (C, *F*_ST_ = 0.21-0.29), (A, *F*_ST_ = 0.16-0.24). Between Syrian/ Lebanese populations and the O lineage the distinction is more subtle (O, *F*_ST_ = 0.07-0.22) by comparison with values within Syria and Lebanon (*F*_ST_ = 0.01- 0.14) (Additional file 1: Figure S3).

## Discussion

Microsatellites have been widely used to study the genetic variation in honeybees [19-28]. In this study we compared bees from Syria and Lebanon and Iraq with those from representative populations in Europe, Africa and the Middle East. To achieve this we amplified 14 microsatellite loci from 2194 individuals in order to characterize evolutionary relationships, population structure and the genetic diversity of Syrian honeybee populations. Our data suggest that *A. m. syriaca* could represent a fifth major genetic lineage of honeybees.

### Genetic diversity of near east populations

Well differentiated from the African and Caucasian populations, Syrian and Lebanese populations were largely cohesive, and values for inter-population *F*_ST_ never rose above 0.02. In terms of genetic diversity, populations were also comparable. Mean heterozygosity per locus and per population range from 0.595 in Al-Quamishli to 0.731 (Nabatiyeh) and are intermediate between those of the A lineage (0.697-0.891) and those of the O lineage (0.615-0.678). Genetic diversity within populations was marginally higher for southern populations, whether from Syria or Lebanon (Additional file 1: Table S1). Enhanced genetic diversity in the South could be related to the intensity and abundance of beekeeping activity, which is certainly higher in the south, and will in turn support a greater effective population size. Heterozygosity in these southern populations is reflected by the higher means of the detected alleles for the fourteen studied loci were (13.71), (13.21) and (11.14) for Quneitra, Daraa and Nabatiyeh populations, respectively, Additional file 1: Table S2.

### Allelic introgression in Northern Syria

On the neighbour-joining tree, this Syria/Lebanon group (Z group) of populations is well separated from the C lineage with a long branch and a high bootstrap value (100%). Nevertheless, the separation of the Syrian populations from the O lineages seems to be less supported with lower bootstrap values. This difficulty of discrimination from the O lineage is probably because the studied region lies in a contact zone between the African continent and the Caucasus, whereby physical proximity drives introgression between the two lineages.

Contact zones in *Apis mellifera* have already been observed on the other side of the Mediterranean sea between M and A lineages for Spanish populations. Gene flow through Gibraltar has allowed allelic exchanges to take place between North African (Algeria and Morocco) and West European honeybee populations over many generations, a phenomenon previously confirmed at molecular level by a north–south gradient of M and A lineages in Spain and Morocco respectively [29].

On the NJ tree, Al-Hasakah and Al-Quamishli populations located in north of Syria are grouped together on a branch clearly separated from the other Syrian populations with high bootstrap value (Figure 3). Such separation is also supported by the PCoA whereby these two populations seem to be intermediates between Syrian and O lineages populations. According to STRUCTURE, the assignments of individuals show clearly an admixture in these populations assigning individuals either to the O group or to the Z group. These two populations are very close and in direct contact with the Southern Turkish border, which can explain a high-level allele admixture between Syrian (Z) and Turkish (O) lineage. Moreover, the mtDNA analysis already shows that these two populations are respectively composed of 91% and 100% of C2 haplotypes, which may be linked to the O lineage [11]. Two other populations (Al-Mawsil, Tartus) seem to be differentiated from the other populations.

Population of Al-Mawsil from Iraq is supposed (according to its geographical location) to belong to *A. m. meda*, a subspecies from the O morphological lineage expected to spread to the North of Iraq [1,2]. This population is indeed in the range of the O lineage according to the PCoA. Nevertheless it is separated from the O lineage group on the NJ tree and clearly separated on the third axe of the PCoA indicating probably that *A. m. meda* can be differentiated from the other O lineages subspecies.

The situation is different for the population of Tartus, which is not geographically close to Al-Mawsil. This population is probably influenced by a gene flow coming from the O lineage through the Turkish border and via Cyprus. Anecdotal information from local beekeepers suggests that multiple colonies have recently been imported via this route. Indeed this population contained 10% of haplotypes C1 and C3 which are characteristic to central European subspecies belonging to the C lineage [11].

### A fifth group of honeybee populations

The Syrian honeybee subspecies *Apis mellifera syriaca* is, geographically speaking, an oriental honeybee and takes, based on classical morphometry, a central position among the four other lineages in Ruttner’s phenogram [2], closest to the African lineage ‘A’. Ruttner’s conclusion concerning *A. m. syriaca* was supported by [11] who grouped this subspecies alongside African individuals. In the current dataset, all clustering algorithms implemented (STRUCTURE, DAPC, a NJ dendrogram as well as the PCoA) suggest the presence of five groups among the data. For STRUCTURE at least, there is evidence for a more fundamental division between Eastern (C + O) and Afro-European populations. The subdivision between old world honeybees is well established, and has been attributed to at least two ‘Out of Africa’ migrations [6,7]. However, our data indicate that the four groups (M, A, C, O) defined in previous studies are likely to represent a simplification. Lebanese and Syrian populations form a fifth group, albeit with some overlap with nearby *A. m. anatoliaca*. The phylogeographic origin of Syrian/Lebanese *A. m. syriaca* is open for debate. In contrast to the nuclear data presented here in which suggest the sister group status between these bees and group O; mtDNA analyses in fact suggest a north-east African origin [11]. As such *A. m. syriaca* fits completely within the mtDNA haplotype Z, a new subgroup of the African evolutionary lineage A described by [11]. Whether such apparent incongruence represents mitochondrial introgression, or an independent African origin for *A. m. syriaca,* remains to be seen, as no North-East African nuclear data is present within the current study (samples Morocco and Algeria only were included).

## Conclusions

In summary, our data suggest that Syrian, Lebanese and Iraqi honeybee populations form a separate fifth major honeybee lineage. Affiliations between *A. m. syriaca* and North African honeybees could suggest an independent African origin for this group. However, *A. m. syriaca* also has affiliations with oriental lineage ‘O’ , and further samples from North Africa are required to clarify the relationship. From a conservation genetic point of view, the presence of an indigenous clade in Syria is encouraging. However, queen importations and allelic introgression imposed by modern beekeeping activities are nonetheless detectable, and our study demonstrates the value of rigorous microsatellite analysis in demarcating population boundaries. For future preservation of the genetic diversity of this locally adapted subspecies we strongly recommend the establishment of genetic conservation centers for the Syrian honeybee *Apis mellifera syriaca*.

## Methods

### Sampling and DNA extraction

**Figure 5:**
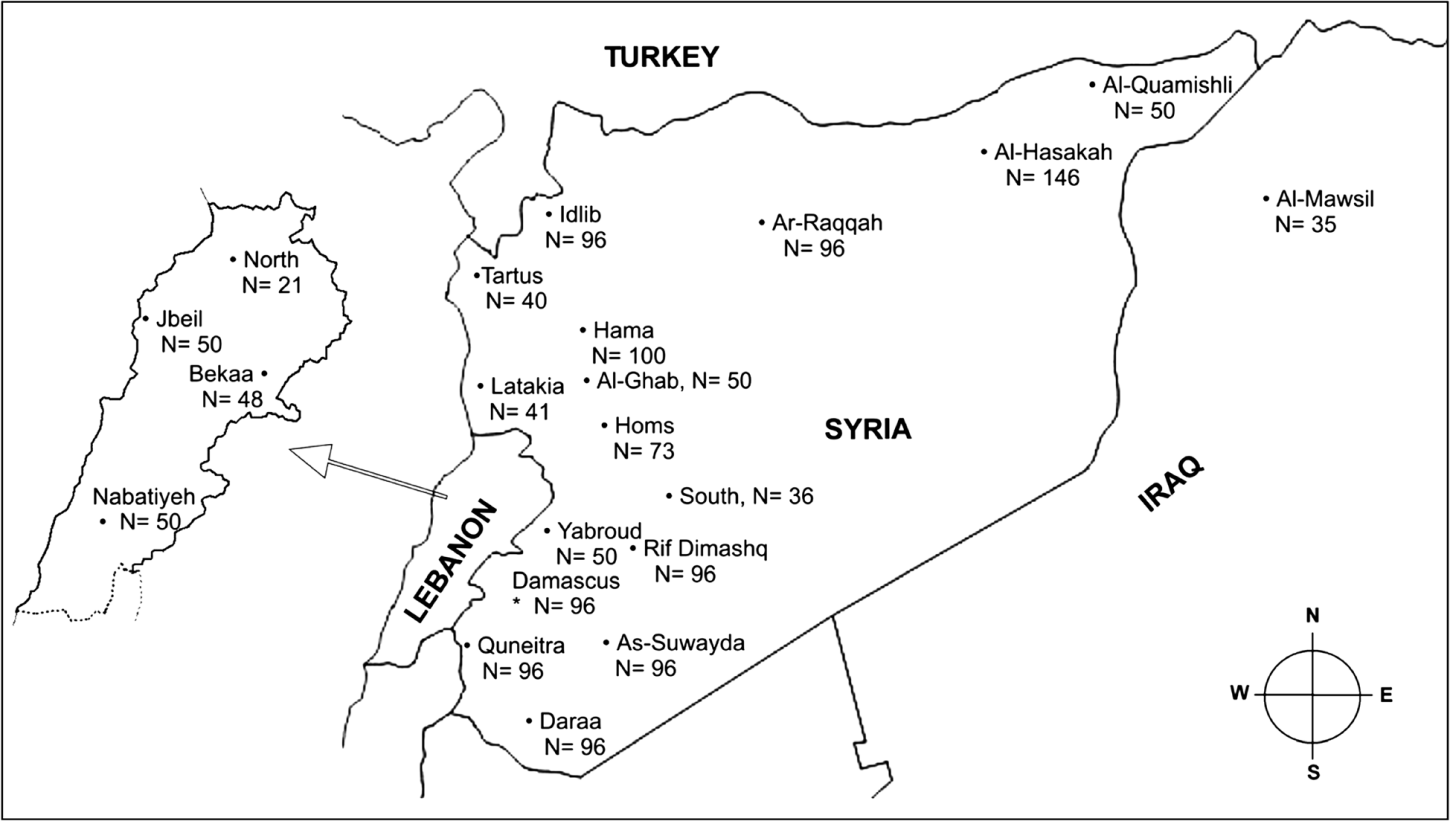
**Shows the geographical locations of the sampled regions in Syria, Lebanon and Iraq.** They comprise 16 populations from Syria, one from Iraq and four from Lebanon. N is the number of sampled honeybees in each population.

Honeybee workers *‘A. m. syriaca’* (N: 1462) were collected in 2007 and 2008 from sixteen Syrian, four Lebanese and one Iraqi study site, sampling one worker per managed hive. Sample numbers were as follows: Syria (Damascus: 96, Rif Dimashq: 96, As-Suwayda: 96, Hama: 100, Daraa: 96, Al-Hasakah: 146, Homs: 73, Idlib: 96, Quneitra: 96, Latakia: 41, Ar-Raqqah: 96, Tartus: 40, Al-Quamishli: 50, Yabroud: 50, Alghab: 50 and South: 36), Lebanon (Bekaa: 48, Jbeil: 50, Nabatiyeh: 50 and North: 21) and Al-Mawsil: 35 from Iraq. The geographical locality of each region and the number of samples is shown in Figure 5. Each worker bee was individually preserved in absolute ethanol, and wings were removed and preserved in ethanol for further morphometrical analysis. DNA was extracted from the thorax using the Chelex method [30] and conserved at -20°C.

### Microsatellite analysis

DNA samples were amplified using multiplex PCR reaction with 14 microsatellite loci. Ten of these microsatellite loci were already known (A7, A28, A113, A43, A88, Ap43, Ap55, Ap81, B24 and B124) [20,23,31-33], while the four others were newly identified from the *Apis* genome [34] and tested for diversity against a representative panel of individuals:

Ap33: Ap33-1 5′-TTTCTTTTTGTGGACAGCG-3′;

Ap33-2 5′-AAATATGGCGAAACGTGTG -3′,

A8: A8-1 5′-CGAAGGTAAGGTAAATGGAAC-3′;

A8-2 5′-GGCGGTTAAAGTTCTGG-3′,

Ap36: Ap36-1 5′-CTACGCGCTTACAGGGCA-3′;

Ap36-2 5′-GCCGAAATTCAACGCTCA-3′,

Ap66: Ap66-1 5′-TTGCATTCGGTCTCCAGC-3′;

Ap66-2 5′-ACTTGCCGCGGTATCTGA-3′.

PCR amplifications were carried out in a total volume of 10 μL, containing 1 μL of Taq 5× buffer (Promega), 1.2 mM MgCl2, 25 pmoles of each primers, 1 μL bovine serum albumin (BSA), 25 nmoles of each dNTP, 0.6 unit of Promega Taq polymerase and 1.0 μL of DNA extract. Annealing temperature was set at 54°C for each plex. PCR products were visualized by capillary electrophoresis (Applied Biosystems 3130) and sized with the internal size-standard ROX from Applied Biosystems. GeneMapper v.4.0 software (Applied Biosystems) was subsequently used for allelic scoring.

Our population samples were compared to fourteen additional populations representing the Ruttner’s evolutionary lineages. Subspecies names used in the current study were given according to the geographical origin of the sampled population and from [1]. Reference populations are: *A. m. mellifera* from France (n = 50) and Belgium (n = 48), as well as *A. m. iberiensis* from Portugal (n = 24) for the (M) lineage; *A. m. intermissa* from Morocco (n = 28) and Algeria (n = 165) for the (A) lineage; *A. m. carnica* from Hungaria (n = 24), Slovenia (n = 91) and Croatia (n = 50) as well as *A. m. cecropia* from Greece (n = 49) for (C) lineage; and finally *A. m. pomonella* from Uzbeckistan (n = 37), Kazakhstan (n = 39), *A. m. caucasica* from Georgia (n = 25), Armenia (n = 17) and *A. m. anatoliaca* from Turkey (n = 24, n = 61) for the Oriental lineage (O).

### Statistical analysis

Identification of genetically similar groups of individuals was obtained with the software STRUCTURE v2.3.3 [35]. The results were based on simulations of 10 000 burn-in steps and MCMC (Markov Chain Monte Carlo algorithm) iterations. The true number of clusters (*K*) was estimated using the value for ∆K [17]. Non-parametric (free from Hardy-Weinberg constraints) population definition was achieved in parallel via *K*-means clustering algorithm. As described in [36]; the ‘true’ number of populations can be defined by reference to the Bayesian Information Criterion (BIC), which reaches a minimum when the best supported assignment of individuals to the appropriate number of clusters is approached. In practice, this number is selected at the ‘elbow’ of the BIC curve. The relationship between these clusters and the individuals within them can be evaluated via a discriminant analysis of principal components (DAPC), again as in [36]. Expected heterozygosity for each locus in each population and number of alleles has been calculated using ARLEQUIN v3.11 software [37]. To evaluate the genetic relationships between populations, a neighbour-joining tree was constructed from microsatellite data using the chord distance of [18]. Bootstrap values were computed over 2000 replications [38] re-sampling individuals within population. The genetic differentiation between populations was computed using unbiased estimated of *F*st values provided by GENEPOP package version 3.1 [39]. Finally, to visualize the groups in a multivariate space, NUEES software (version 0.8) was also used to perform Principal Component Analyses (PCoA) using the distance matrix between population [18] and between individuals DAS from [40].

### Availability of supporting data

The data sets supporting the results of this article are available at http://www.labarchives.com/

https://mynotebook.labarchives.com/share/Alburaki/My45fDIzMzUzLzMvVHJlZU5vZGUvMjYyMjU1MDg2OXw5Ljk=

## Competing interests

The authors declare that they have no competing interests.

## Authors’ contributions

MA carried out data collection and analysis. BB and HL carried out data analysis. SM participated in data collection. LG participated in the design of the study and coordination. SWS participated in discussion of the study. AA participated in the design of the study and sample collection. LG participated in data discussion and manuscript preparation. MA participated in the design of the study, data analysis and manuscript preparation. All authors read and approved the final manuscript.

## Supplementary Material

Additional file 1: Figure S1 Genetic clustering of few Syrian, Lebanese and one Iraqi honeybee populations and five honeybee subspecies using 14 microsatellite loci. The number of cluster K was set at five populations for the Syrian and Lebanese populations. **Figure S2.** Output of the discriminant analysis of principal components (DAPC) for the studied populations form Syria, Lebanon and Iraq along with the four populations of the reference lineages (M, A, C O). **Figure S3.** Pairwise multilocus unbiased estimate of *F*st calculated by ARLEQUIN software in each studied and reference populations. Reference populations are representing the four evolutionary lineages M, A, C and O. **Table S1.** Expected heterozygosity (H_E_) for each locus in each studied populations as well as overall loci for each studied populations. Refer to Table 1 for the abbreviations. **Table S2.** Number of detected alleles for each microsatellite locus in each studied population. Mean values and Standard deviations (s.d.) are calculated for each population. Refer to Table 1 for the abbreviations. Click here for file
