# Supplementary material for: A fifth major genetic group among honeybees revealed in Syria
Source: BMC Genet. 2013 Dec 6;14:117. doi: 10.1186/1471-2156-14-117 (PMC4029286; doi:10.1186/1471-2156-14-117)
Supplement: Additional file 1: Figure S1 — Genetic clustering of few Syrian, Lebanese and one Iraqi honeybee populations and five honeybee subspecies using 14 microsatellite loci. The number of cluster K was set at five populations for the Syrian and Lebanese populations. Figure S2. Output of the discriminant analysis of principal components (DAPC) for the studied populations form Syria, Lebanon and Iraq along with the four populations of the reference lineages (M, A, C O). Figure S3. Pairwise multilocus unbiased estimate of Fst calculated by ARLEQUIN software in each studied and reference populations. Reference populations are representing the four evolutionary lineages M, A, C and O. Table S1. Expected heterozygosity (HE) for each locus in each studied populations as well as overall loci for each studied populations. Refer to Table 1 for the abbreviations. Table S2. Number of detected alleles for each microsatellite locus in each studied population. Mean values and Standard deviations (s.d.) are calculated for each population. Refer to Table 1 for the abbreviations. [file 1471-2156-14-117-S1.docx]

**Supplementary Material:**

**Figure S1**: Genetic clustering of few Syrian, Lebanese and one Iraqi honey bee populations and five honey bee subspecies using 14 microsatellite loci. The number of cluster K was set at five populations for the Syrian and Lebanese populations.


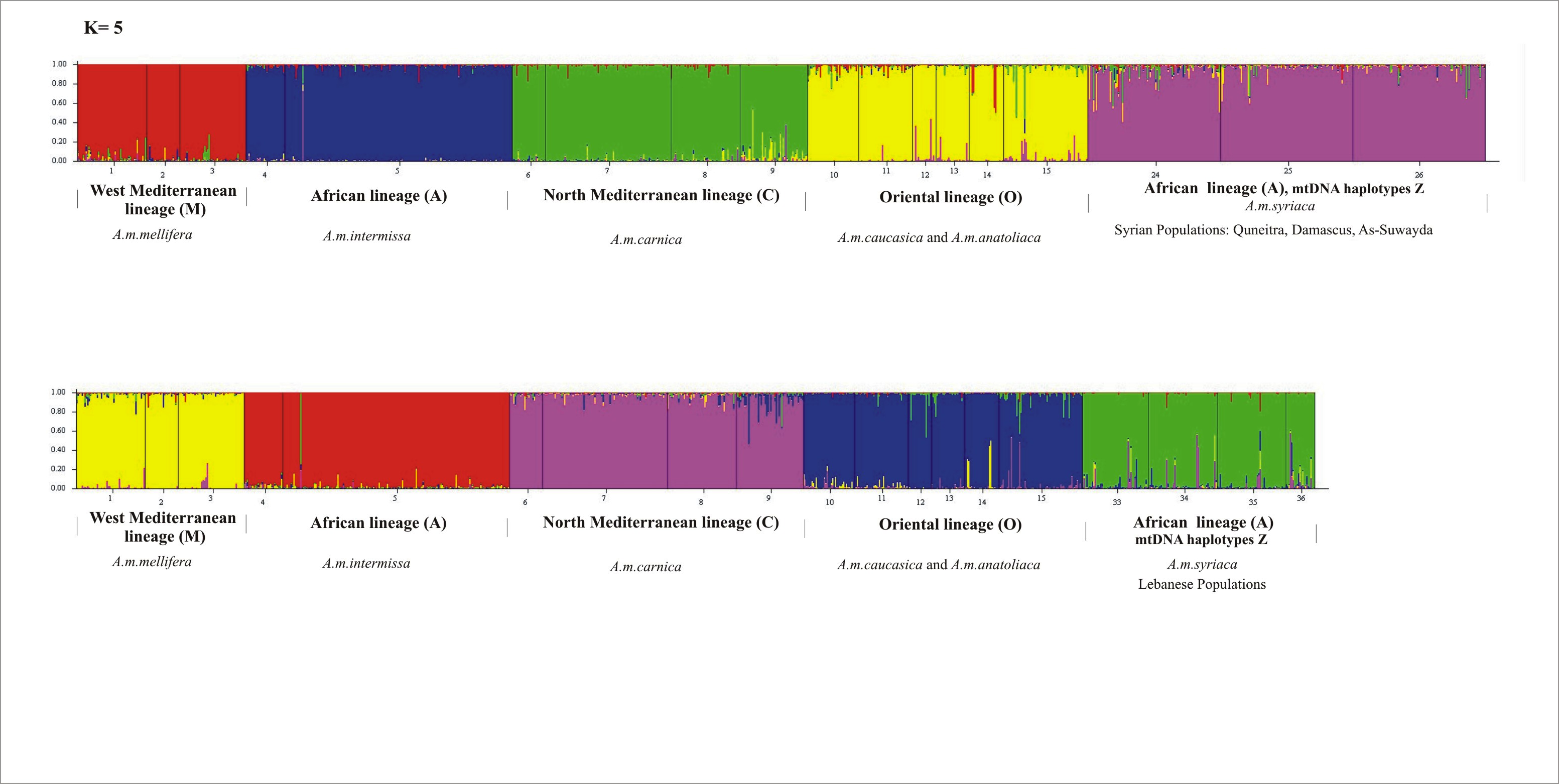
**Figure S2**: Output of the discriminant analysis of principal components (DAPC) for the studied populations form Syria, Lebanon and Iraq along with the four populations of the reference lineages (M, A, C O).

**
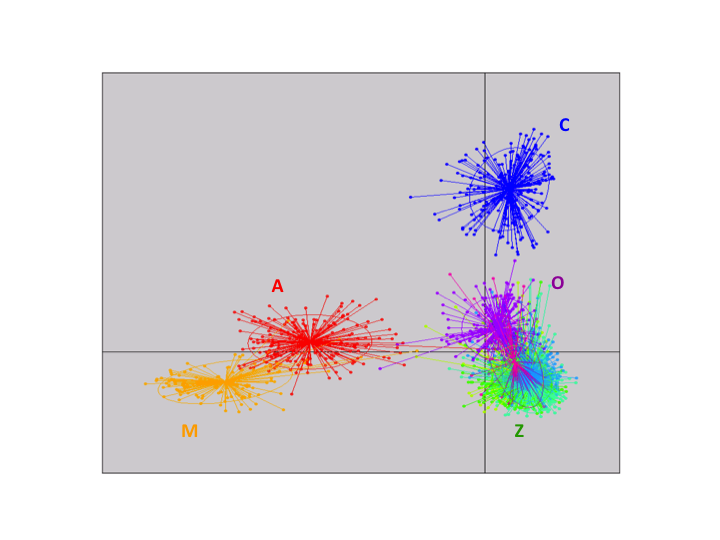
**

**Figure S3**: Pairwise multilocus unbiased estimate of *F*st calculated by ARLEQUIN software in each studied and reference populations. Reference populations are representing the four evolutionary lineages M, A, C and O.


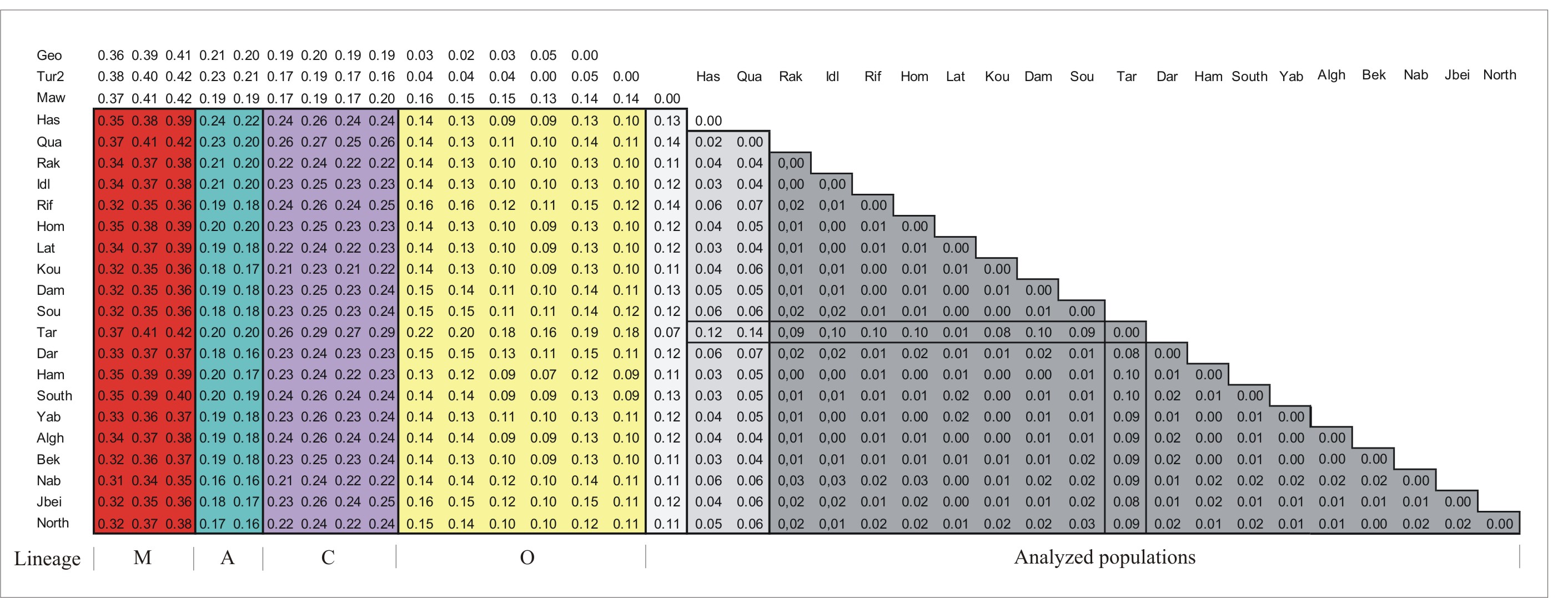


**Table S1:** Expected heterozygosity (H_E_) for each locus in each studied populations as well as overall loci for each studied populations. Refer to Tab. 1 for the abbreviations.

| Locus | Maw | Has | Qua | Rak | Idl | Rif | Hom | Lat | Kou | Dam | Sou |
| --- | --- | --- | --- | --- | --- | --- | --- | --- | --- | --- | --- |
| B124 | 0,817 | 0,525 | 0,570 | 0,494 | 0,466 | 0,435 | 0,472 | 0,466 | 0,582 | 0,553 | 0,481 |
| A43 | 0,737 | 0,720 | 0,747 | 0,844 | 0,794 | 0,869 | 0,817 | 0,794 | 0,863 | 0,842 | 0,862 |
| A88 | 0,612 | 0,502 | 0,557 | 0,680 | 0,585 | 0,661 | 0,649 | 0,642 | 0,775 | 0,565 | 0,736 |
| A24 | 0,655 | 0,549 | 0,503 | 0,555 | 0,540 | 0,556 | 0,537 | 0,552 | 0,607 | 0,583 | 0,669 |
| Ap33 | 0,943 | 0,837 | 0,832 | 0,868 | 0,874 | 0,896 | 0,884 | 0,905 | 0,904 | 0,912 | 0,893 |
| A88 | 0,784 | 0,488 | 0,524 | 0,594 | 0,571 | 0,586 | 0,517 | 0,632 | 0,636 | 0,638 | 0,566 |
| A113 | 0,813 | 0,798 | 0,747 | 0,765 | 0,816 | 0,852 | 0,823 | 0,811 | 0,858 | 0,797 | 0,795 |
| Ap43 | 0,790 | 0,749 | 0,727 | 0,697 | 0,797 | 0,767 | 0,760 | 0,833 | 0,758 | 0,704 | 0,721 |
| A28 | 0,137 | 0,113 | 0,059 | 0,193 | 0,300 | 0,582 | 0,258 | 0,394 | 0,415 | 0,455 | 0,507 |
| A7 | 0,914 | 0,910 | 0,874 | 0,945 | 0,932 | 0,928 | 0,937 | 0,944 | 0,943 | 0,936 | 0,929 |
| Ap36 | 0,817 | 0,834 | 0,802 | 0,817 | 0,784 | 0,804 | 0,814 | 0,746 | 0,780 | 0,804 | 0,782 |
| Ap55 | 0,709 | 0,557 | 0,429 | 0,743 | 0,703 | 0,682 | 0,722 | 0,697 | 0,732 | 0,645 | 0,681 |
| Ap81 | 0,463 | 0,359 | 0,401 | 0,530 | 0,496 | 0,557 | 0,509 | 0,587 | 0,473 | 0,615 | 0,610 |
| Ap66 | 0,465 | 0,524 | 0,563 | 0,571 | 0,574 | 0,520 | 0,661 | 0,581 | 0,600 | 0,554 | 0,553 |
| Mean | 0,690 | 0,604 | 0,595 | 0,664 | 0,659 | 0,693 | 0,669 | 0,685 | 0,709 | 0,686 | 0,699 |
| s.d. | 0,215 | 0,218 | 0,216 | 0,194 | 0,181 | 0,159 | 0,190 | 0,163 | 0,161 | 0,149 | 0,143 |

| Locus | Tar | Dar | Ham | South | Yab | Algh | Bek | Nab | Jbei | North | Mean | s.d. | Tot. Het. |
| --- | --- | --- | --- | --- | --- | --- | --- | --- | --- | --- | --- | --- | --- |
| B124 | 0,492 | 0,457 | 0,534 | 0,448 | 0,410 | 0,522 | 0,588 | 0,535 | 0,422 | 0,517 | 0,630 | 0,162 | 0,733 |
| A43 | 0,606 | 0,837 | 0,862 | 0,757 | 0,886 | 0,842 | 0,867 | 0,833 | 0,854 | 0,851 | 0,655 | 0,232 | 0,857 |
| A88 | 0,484 | 0,686 | 0,615 | 0,664 | 0,608 | 0,578 | 0,611 | 0,768 | 0,672 | 0,633 | 0,563 | 0,182 | 0,729 |
| A24 | 0,813 | 0,654 | 0,565 | 0,548 | 0,608 | 0,611 | 0,583 | 0,599 | 0,659 | 0,627 | 0,555 | 0,125 | 0,694 |
| Ap33 | 0,885 | 0,897 | 0,856 | 0,915 | 0,894 | 0,878 | 0,914 | 0,909 | 0,913 | 0,880 | 0,884 | 0,027 | 0,922 |
| A88 | 0,656 | 0,634 | 0,579 | 0,561 | 0,604 | 0,565 | 0,608 | 0,674 | 0,678 | 0,599 | 0,546 | 0,141 | 0,703 |
| A113 | 0,889 | 0,834 | 0,805 | 0,856 | 0,807 | 0,836 | 0,856 | 0,833 | 0,817 | 0,857 | 0,749 | 0,177 | 0,881 |
| Ap43 | 0,749 | 0,815 | 0,699 | 0,619 | 0,753 | 0,716 | 0,745 | 0,895 | 0,806 | 0,823 | 0,767 | 0,097 | 0,833 |
| A28 | 0,288 | 0,510 | 0,264 | 0,207 | 0,434 | 0,403 | 0,282 | 0,567 | 0,546 | 0,380 | 0,322 | 0,200 | 0,502 |
| A7 | 0,955 | 0,938 | 0,942 | 0,934 | 0,936 | 0,931 | 0,935 | 0,908 | 0,923 | 0,925 | 0,836 | 0,213 | 0,953 |
| Ap36 | 0,760 | 0,792 | 0,855 | 0,842 | 0,814 | 0,745 | 0,775 | 0,796 | 0,789 | 0,699 | 0,754 | 0,135 | 0,839 |
| Ap55 | 0,705 | 0,720 | 0,686 | 0,693 | 0,695 | 0,673 | 0,624 | 0,656 | 0,582 | 0,660 | 0,673 | 0,078 | 0,749 |
| Ap81 | 0,672 | 0,529 | 0,426 | 0,511 | 0,601 | 0,551 | 0,589 | 0,584 | 0,577 | 0,621 | 0,471 | 0,160 | 0,559 |
| Ap66 | 0,632 | 0,615 | 0,646 | 0,620 | 0,635 | 0,631 | 0,604 | 0,670 | 0,637 | 0,693 | 0,545 | 0,148 | 0,715 |
| Mean | 0,685 | 0,708 | 0,667 | 0,655 | 0,692 | 0,677 | 0,684 | 0,731 | 0,705 | 0,698 | 0,639 | 0,089 | 0,762 |
| s.d. | 0,181 | 0,150 | 0,189 | 0,200 | 0,164 | 0,154 | 0,176 | 0,134 | 0,149 | 0,154 | 0,196 | 0,050 | 0,125 |

**Table S2**: Number of detected alleles for each microsatellite locus in each studied population. Mean values and Standard deviations (s.d.) are calculated for each population. Refer to Tab. 1 for the abbreviations.

|  |  |  |  |  |  |  |  |  |  |  | |  |  | |  |
| --- | --- | --- | --- | --- | --- | --- | --- | --- | --- | --- | --- | --- | --- | --- | --- |
|  |  |  |  |  |  |  |  |  |  |  | |  |  | |  |
| Locus | Maw | Has | Qua | Rak | Idl | Rif | Hom | Lat | Kou | Dam | Sou | | |  |  |
| B124 | 11 | 5 | 5 | 8 | 6 | 7 | 7 | 5 | 11 | 7 | 8 | | |  |  |
| A43 | 5 | 8 | 5 | 13 | 13 | 15 | 13 | 9 | 16 | 15 | 15 | | |  |  |
| A88 | 4 | 6 | 3 | 9 | 10 | 9 | 9 | 8 | 12 | 8 | 12 | | |  |  |
| A24 | 11 | 6 | 2 | 8 | 7 | 7 | 5 | 5 | 8 | 8 | 8 | | |  |  |
| Ap33 | 16 | 14 | 9 | 16 | 14 | 14 | 14 | 15 | 17 | 17 | 14 | | |  |  |
| A88 | 5 | 4 | 3 | 7 | 4 | 4 | 6 | 6 | 7 | 7 | 7 | | |  |  |
| A113 | 11 | 10 | 10 | 11 | 11 | 13 | 13 | 12 | 14 | 14 | 12 | | |  |  |
| Ap43 | 13 | 21 | 11 | 17 | 25 | 24 | 22 | 21 | 26 | 22 | 26 | | |  |  |
| A28 | 3 | 7 | 4 | 4 | 5 | 6 | 3 | 5 | 8 | 6 | 6 | | |  |  |
| A7 | 19 | 24 | 17 | 26 | 23 | 30 | 26 | 24 | 26 | 27 | 26 | | |  |  |
| Ap36 | 13 | 17 | 14 | 13 | 13 | 18 | 16 | 13 | 18 | 17 | 16 | | |  |  |
| Ap55 | 5 | 6 | 4 | 7 | 8 | 10 | 9 | 7 | 12 | 9 | 9 | | |  |  |
| Ap81 | 9 | 7 | 6 | 9 | 10 | 11 | 10 | 7 | 9 | 10 | 10 | | |  |  |
| Ap66 | 7 | 5 | 5 | 4 | 4 | 5 | 5 | 4 | 8 | 4 | 6 | | |  |  |
| Mean | 9,43 | 10,00 | 7,00 | 10,86 | 10,93 | 12,36 | 11,29 | 10,07 | 13,71 | 12,21 | 12,50 | | |  |  |
| s.d. | 4,83 | 6,43 | 4,52 | 5,88 | 6,46 | 7,49 | 6,64 | 6,23 | 6,29 | 6,69 | 6,56 | | |  |  |
|  |  |  |  |  |  |  |  |  |  |  | |  |  | |  |
|  |  |  |  |  |  |  |  |  |  |  | |  |  | |  |
| Locus | Tar | Dar | Ham | South | Yab | Algh | Bek | Nab | Jbei | North | | Mean | s.d. | | Tot. N. |
| B124 | 5 | 9 | 8 | 5 | 5 | 4 | 8 | 9 | 7 | 4 | | 8,61 | 3,38 | | 22 |
| A43 | 9 | 17 | 13 | 10 | 14 | 11 | 16 | 13 | 13 | 8 | | 9,14 | 4,52 | | 33 |
| A88 | 6 | 9 | 10 | 7 | 7 | 7 | 7 | 8 | 7 | 4 | | 6,39 | 2,65 | | 26 |
| A24 | 14 | 8 | 12 | 6 | 8 | 6 | 6 | 8 | 8 | 7 | | 6,03 | 2,83 | | 30 |
| Ap33 | 16 | 16 | 16 | 15 | 15 | 13 | 15 | 16 | 16 | 15 | | 14,33 | 2,04 | | 28 |
| A88 | 7 | 5 | 5 | 4 | 4 | 4 | 5 | 6 | 6 | 6 | | 5,28 | 1,50 | | 14 |
| A113 | 14 | 12 | 12 | 12 | 11 | 10 | 14 | 11 | 10 | 10 | | 10,83 | 2,65 | | 23 |
| Ap43 | 15 | 30 | 21 | 16 | 17 | 15 | 16 | 22 | 20 | 11 | | 16,81 | 6,33 | | 46 |
| A28 | 4 | 8 | 4 | 4 | 7 | 5 | 5 | 5 | 9 | 5 | | 4,72 | 2,46 | | 18 |
| A7 | 24 | 26 | 25 | 21 | 24 | 24 | 20 | 23 | 22 | 15 | | 19,25 | 7,41 | | 63 |
| Ap36 | 15 | 20 | 17 | 13 | 14 | 13 | 13 | 13 | 14 | 10 | | 13,31 | 3,23 | | 29 |
| Ap55 | 8 | 9 | 8 | 7 | 6 | 6 | 5 | 8 | 7 | 6 | | 7,58 | 2,10 | | 25 |
| Ap81 | 8 | 10 | 8 | 5 | 7 | 7 | 6 | 9 | 8 | 4 | | 6,69 | 2,90 | | 19 |
| Ap66 | 4 | 6 | 6 | 5 | 5 | 4 | 4 | 5 | 5 | 4 | | 5,67 | 2,39 | | 25 |
| Mean | 10,64 | 13,21 | 11,79 | 9,29 | 10,29 | 9,21 | 10,00 | 11,14 | 10,86 | 7,79 | | 9,61 | 2,27 | | 29 |
| s.d. | 5,81 | 7,61 | 6,15 | 5,33 | 5,78 | 5,65 | 5,39 | 5,75 | 5,35 | 3,89 | | 5,37 | 1,23 | | 12 |
